# Finite Element Simulation of Hot Rolling for Large-Scale AISI 430 Ferritic Stainless-Steel Slabs Using Industrial Rolling Schedules—Part 2: Simulation of the Roughing Stage and Comparison with Experimental Results

**DOI:** 10.3390/ma18061298

**Published:** 2025-03-15

**Authors:** Adrián Ojeda-López, Marta Botana-Galvín, Juan F. Almagro Bello, Leandro González-Rovira, Francisco Javier Botana

**Affiliations:** 1 Department of Materials Science and Metallurgical Engineering and Inorganic Chemistry, Faculty of Sciences, University of Cadiz, Campus Río San Pedro S/N, 11510 Puerto Real, Spain; adrian.ojeda@uca.es (A.O.-L.); leandro.gonzalez@uca.es (L.G.-R.); 2 Titania Ensayos y Proyectos Industriales, Edificio RETSE, Nave 4, Parque Tecnobahía, 11500 El Puerto de Santa María, Spain; marta.botana@titania.aero; 3 Laboratory & Research Section, Technical Department, Acerinox Europa S.A.U., 11379 Los Barrios, Spain; juan.almagro@acerinox.com

**Keywords:** hot rolling, flat rolling, stainless steel, AISI 430, numerical simulation, finite element method

## Abstract

Modeling hot rolling remains a major challenge in computational solid mechanics. It demands the simultaneous consideration of geometric and material responses. Although the finite element method (FEM) is widely used, multi-pass simulations often treat each pass independently, leading to error accumulation, particularly in flat product rolling, where inter-pass interactions are crucial. Advanced models and remeshing techniques have been developed to address these issues, but substantial computational resources are required. In this study, a previously validated and simplified 3D FEM model was employed to simulate the initial stages of the hot rolling of large-scale AISI 430 ferritic stainless-steel slabs, using data from an industrial rolling schedule. Specifically, the simulations encompassed preheating and descaling, and seven passes of the roughing stage. Through these simulations, a transfer bar with an approximate length of 16,100 mm was obtained. The simulated thickness and rolling load values were compared with experimental data, demonstrating good agreement in most passes. Subsequently, the temperature, effective plastic strain, and equivalent stress distributions along the rolled material were extracted and analyzed. The results highlighted that the employed model adequately predicted the variations in the analyzed parameters throughout the volume of the rolled material during the different stages of the process. However, discrepancies were identified in the rolling load values during the final passes, which were attributed to the presence of phenomena not considered in the constitutive model used. This model will be refined in future studies to reduce the error in the rolling load estimation.

## 1. Introduction

Computational solid mechanics remains one of the most challenging research areas in engineering, particularly when addressing large deformation problems such as hot rolling [1]. Hot rolling is inherently a complex three-dimensional problem, requiring the simultaneous consideration of geometric and material responses [2]. Despite significant advances in physical modeling, material property characterization, computational power, and numerical methods [3], accurately simulating the hot rolling process remains a complex task. The finite element method (FEM) [4] is widely employed in these simulations; however, its application to multi-pass rolling presents unique challenges.

In many studies, successive passes in rolling simulations are treated independently, which can lead to the accumulation of errors that compromise the fidelity of the overall simulation [5,6,7]. This approach is particularly problematic in processes such as the hot rolling of flat products, where the interaction between consecutive passes plays a critical role. To mitigate error accumulation, advanced mathematical models for multi-pass rolling have been proposed [8]. These models not only need to address algorithmic challenges—such as the rebuilding of the finite-element grid, the transfer of metal properties between passes, and the automation and analysis of computations—but also must accurately capture the non-monotonic nature of deformation, including strain hardening and the subsequent removal effects during and between passes [9].

Moreover, the simulation of three-dimensional large-deformation processes with FEM is further complicated by mesh distortion. Severe deformations inherent in hot rolling can lead to a significant loss of accuracy, as distorted meshes adversely affect the quality of the numerical results [10]. To overcome this, various remeshing techniques have been developed and integrated into commercial FEM software, with methods such as the arbitrary Lagrangian–Eulerian approach proving effective in maintaining mesh quality and ensuring consistency between numerical and experimental outcomes [11]. Additionally, the spatial resolution required to accurately simulate these processes often demands substantial computational resources. This, combined with the limitations imposed by computer hardware and simulation software, results in longer processing times and significant memory allocation [12]. As a result, in order to simulate rolling processes, it is necessary to introduce considerable simplifications into the models, which in extreme cases means that 2D models are used. Moreover, simulations are typically based on small-sized specimens and modified rolling schedules. Consequently, these models often do not accurately represent the complex, large-scale processes implemented in industrial practice.

In [13], we proposed the design and optimization of a 3D FEM model to simulate the hot rolling of large-format AISI 430 ferritic stainless-steel slabs. In that study, the optimization of the model was based on the simulation of the first pass of the process. The objective of the present study was to simulate the seven passes that constitute the roughing stage of the hot rolling process for large-format slabs of AISI 430 ferritic stainless steel, using an industrial rolling schedule. The applicability of the model was examined for slabs with initial dimensions of 2000 mm × 1280 mm × 200 mm. The inherent complexities of consecutive pass simulations were addressed through the integration of remeshing strategies. The results obtained allowed us to estimate the variations in the rolled steel thickness and in the rolling load with the number of passes. The validity of the model was analyzed by comparing the simulated and experimental values of both parameters. Additionally, the results for temperature, effective plastic strain, and equivalent stress were analyzed, and their distributions throughout the rolled material were studied.

In the present work, a methodology is proposed for simulating the entire roughing stage of a multi-pass hot rolling process applied to large-scale stainless-steel slabs. An industrial rolling schedule was utilized, focusing on a material of significant industrial interest, AISI 430 ferritic stainless steel. Advanced remeshing strategies were implemented to prevent mesh distortion, with the mesh being dynamically adapted in each pass to correspond with the evolving thickness of the rolled product, thereby maintaining an adequate resolution in the thickness direction. Unlike previous studies that were limited to laboratory-scale experiments, two-dimensional models, or small specimens, the present work addresses an industrial case, allowing for the accurate simulation of material behavior during the process. Consequently, the proposed methodology enables the extraction of key parameters that allow for a deeper understanding of the industrial process under study, as well as the material behavior. In addition, the proposed methodology could be used to simulate the rolling of any other stainless steel, by using the appropriate values of material characterization and rolling process parameters.

## 2. Materials and Methods

### 2.1. Experimental Procedure

The manufacture of flat products made of AISI 430 ferritic stainless steel, conducted at the facilities of Acerinox Europa S.A.U. (Los Barrios, Spain), consists of two stages. The first stage corresponds to hot rolling, which is followed by cold rolling. The hot rolling process itself is divided into a roughing stage and a hot finishing stage. Before hot rolling, the material undergoes a preheating stage, followed by a descaling treatment.

**Figure 1:**
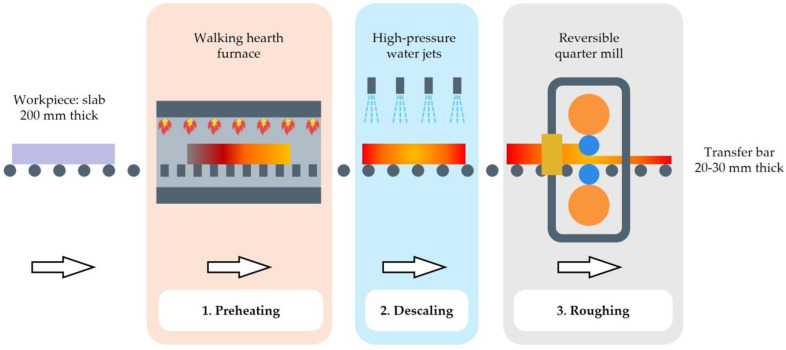
Schematic representation of the initial phases of the hot rolling of AISI 430 stainless-steel slabs.

This study examined the simulation of the preheating, descaling, and roughing stages, which are represented schematically in Figure 1. The analysis focused on an industrial rolling schedule consisting of seven passes, starting from a slab of 12,000 mm × 1280 mm × 200 mm.

The rolling process begins with the preheating phase, as shown in Figure 1(1). During this phase, the slab is placed in a walking hearth furnace until the material reaches a uniform temperature of approximately 1150 °C throughout its volume. After the slab exits the furnace, the descaling phase commences, where high-pressure water jets are used to remove oxide scale, as shown in Figure 1(2). The slab is subsequently reduced in thickness in a reversible quarter mill, as shown in Figure 1(3), by applying seven passes.

**Figure 2:**
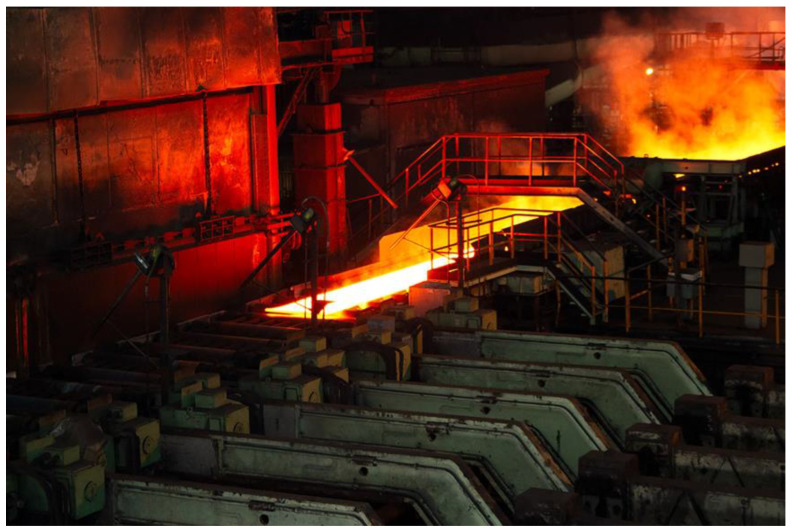
Reversible quarter mill used in the roughing stage under study. Photograph provided by Acerinox Europa S.A.U.

**Figure 3:**
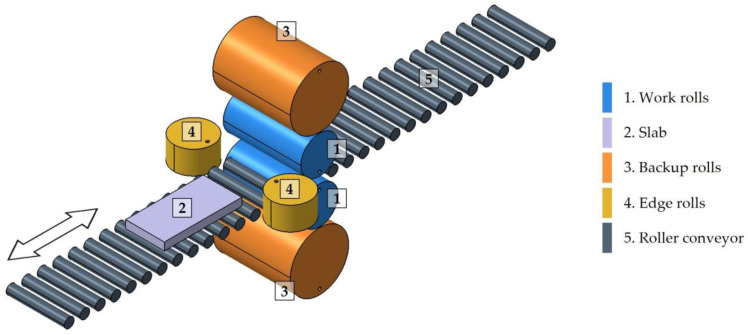
A schematic model of a four high reversible rolling mill used for the roughing stage of the manufacture of AISI 430 ferritic stainless-steel slabs. The arrow indicates the rolling direction. Reprinted with permission from [13], 2024, MDPI.

Figure 2 shows a photograph of the rolling mill under study. A schematic representation of this mill, previously introduced in [13], is also shown in Figure 3. This mill is equipped with two work rolls (Figure 3(1)), through which the material being rolled is passed (Figure 3(2)). The backup rolls (Figure 3(3)) have a larger diameter and are responsible for transmitting the stress to the work rolls. Furthermore, the mill includes two edge rolls (Figure 3(4)), which operate during specific passes to ensure good-quality edges on the rolled product and maintain the slab width within the required tolerances. Additionally, the mill is equipped with a roller conveyor (Figure 3(5)), which facilitates the transportation of the slab during the rolling process.

In the rolling schedule under study, the thickness reduction is achieved by passing the material through the work rolls in seven passes. During the first three passes, secondary descaling is performed. Table 1 provides the average values of the key parameters used in the hot rolling schedule under study. Specifically, this table presents average values for the initial thickness, the applied reduction ratio, the work-roll gap, the edge-roll gap, and the linear speed in each pass.

The rolled material obtained at the exit of the roughing mill is known as the transfer bar. This product has a thickness between 20 mm and 30 mm and constitutes the raw material for the hot finishing stage, the simulation of which was not addressed in this study. In the hot finishing stage, the thickness of the transfer bar is progressively reduced until sheets with a width of 1200 mm and a thickness of 3.5 mm are obtained. These sheets are then coiled, resulting in what is commonly known as black coil.

### 2.2. Material

The material analyzed was AISI 430/EN 1.4016 ferritic stainless-steel quality ACX490, produced by Acerinox Europa S.A.U. This stainless steel is characterized by containing 16.7% Cr, 0.41% Si, 0.37% Mn, and 0.22% Ni as the major alloying elements. The complete composition can be found in [13].

Table 2 provides the values of the main mechanical and thermal properties of AISI 430 at various temperatures close to the working conditions. These values, obtained from experimental data and provided by Acerinox Europa S.A.U., were used as input data in the material’s characterization for the simulations.

### 2.3. FEM Simulation

#### 2.3.1. Numerical Setup and Assumptions

As mentioned previously, this study covers the simulation of the entire roughing stage of the hot rolling process for AISI 430 ferritic stainless-steel slabs. Specifically, the phases of preheating, descaling, and multi-pass rolling were simulated.

The finite element simulation was conducted using the FEM-based software Simufact Forming 2024.2, developed by Hexagon AB (Stockholm, Sweden). This software is widely utilized in the numerical analysis of rolling processes due to its ability to handle complex nonlinear calculations [14]. The direct multi-process solver Pardiso Direct Sparse from Marc^®^, developed by Hexagon AB (Stockholm, Sweden), was employed, with parallel computing implemented on 4 cores.

All the simulations were performed on a workstation equipped with 64 GB of RAM and an Intel^®^ Core™ i7-7700K processor, developed by Intel Corporation (Santa Clara, California, United States), which contains 4 cores and 8 threads, and operates at a maximum frequency of 4.50 GHz.

The rolling process was modeled using a 3D thermo-mechanical coupled finite element approach developed and optimized in [13]. For the optimization of the model, in [13], the rolls were assumed to be rigid bodies due to their significantly higher stiffness compared to the workpiece. On the other hand, the slab was modeled as a temperature-dependent viscoplastic material with isotropic thermal and mechanical properties. In this study, friction between the rolls and the slab was also considered, and a constant value was defined for each pair of contact surfaces. Additionally, thermal effects were included in the thermo-mechanical calculations, defining the heat transfer coefficients (HTCs) as well as the temperatures of the bodies and the surrounding environment.

Based on the results obtained in [13], an optimized model was developed, incorporating the following approximations:

1. Both the geometry and the boundary conditions of the studied process are symmetric with respect to the midplane that divides the width of the workpiece into two equal parts. Therefore, only half of the slab was simulated, with a width of 640 mm. This approach allowed the omission of one edge roll and a reduction in the dimensions of the remaining rolls.
2. At the beginning of the rolling process, the slab is moved by the action of a pusher and slides over a roller conveyor composed of roller sections in the form of quarter cylinders. Upon reaching the edge roll and the work rolls, the slab is gripped, and the pusher ceases to operate. From this point onward, the slab advances due to the action of the rolls.
3. The friction between the roller conveyor and the slab, as well as between the pusher and the slab, is zero.
4. Oxide scale formation is not considered in the simulation.
5. The sides of the workpiece are free surfaces, and the external tractions on the slab surface are zero.

#### 2.3.2. Model Geometry and Mesh Characteristics

**Figure 4:**
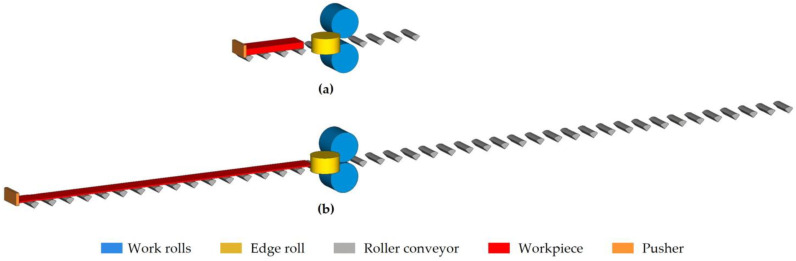
Model of the rolling mill used in the finite element simulations of the roughing stage of AISI 430 stainless-steel slabs. (**a**) Model of the first pass with nine quarter rolls on the roller conveyor. (**b**) Model of the seventh pass with forty-two quarter rolls on the roller conveyor.

The simulations were performed using the validated and optimized model presented in Part 1 [13] of this article, which is illustrated in Figure 4. This model consists of two work rolls, an edge roll, a roller conveyor, the workpiece, and a pusher. The number of quarter rolls on the roller conveyor was variable and was adjusted to the length of the workpiece, ranging from nine in the simulation of the first pass, Figure 4a, to forty-two in the seventh, Figure 4b.

The geometries of the roughing mill and the slab were generated in Blender^®^ 4.1, developed by the Blender Foundation (Amsterdam, The Netherlands), and exported as STL files. Subsequently, these files were imported into Simufact Forming.

To simulate the rolling process, a Lagrangian formulation was employed, which allows for tracking the motion of the material particles throughout their evolution over time. This approach is well suited to accurately capturing plastic deformations and thermal variations occurring during the process. Additionally, a remeshing condition was implemented to prevent mesh distortion due to the deformations that occur during rolling. This approach ensured that the mesh effectively adapted to the changing geometry of the slab, maintaining both its integrity and the accuracy of the obtained results.

A mesh consisting of 4000 eight-node thermally coupled hexahedral elements with an initial element size of 40 mm was assigned to the slab. The mesh was generated using the Sheetmesh mesher included in Simufact Forming, which allows for the creation of uniform meshes distributed in sheets, with a specified number of elements for the thickness. As indicated in [13], the slab mesh was generated using five elements in the thickness direction. This condition was maintained throughout the seven simulated passes, resulting in a reduction in the element size of the workpiece with each pass.

Meanwhile, the rolls and the pusher were assigned a mesh composed of 10,575 four-node, thermally coupled tetrahedral elements with an element size of 50 mm. Thus, at the beginning of the simulation, the mesh was discretized into a total of 14,575 finite elements. Throughout the rolling passes, the total number of elements increased due to the continuous refinement of the slab mesh, which occurred as a result of element size reduction driven by the thickness reduction. Table 3 presents the main parameters of the initial mesh for the slab, as well as the mesh for the rolls and the pusher.

#### 2.3.3. Material Model and Properties

The behavior of AISI 430 ferritic stainless steel was modeled using a temperature-dependent viscoplastic constitutive model, specifically the GMT analytical model, extracted from the material library of Simufact Forming. This model accounts for both the strain hardening and thermal softening effects typically observed in hot rolling processes. The flow stress ($\sigma_{f}$) was determined using Equation (1), which incorporates the effects of strain, strain rate, and temperature, as specified in [13].

(1) $$\sigma_{f}=5862.37\cdot e^{-0.00469\cdot T}\cdot \varphi^{-0.000302\cdot T+0.366}\cdot e^{\frac{-0.0000878\cdot T+0.0724}{\varphi }}\cdot {\dot{\varphi }}^{0.000196\cdot T-0.0547}$$

where

$\sigma_{f}$: Flow stress, [N·m^−2^].

φ: Strain, [Dimensionless].

$\dot{\varphi }$: Strain rate, [s^−1^].

*T*: Temperature, [°C].

**Figure 5:**
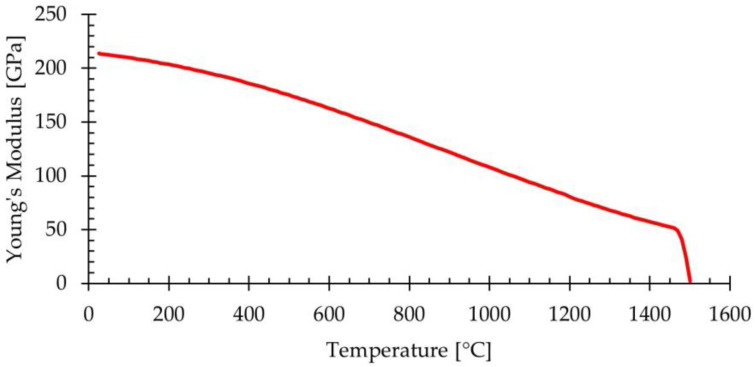
Young’s modulus versus temperature for AISI 430 ferritic stainless steel. Extracted from Simufact Materials 2024.2, developed by Hexagon AB (Stockholm, Sweden).

The material properties used in the simulations are provided in Table 2. The values of Young’s modulus as a function of temperature were obtained from the material library of Simufact Forming for AISI 430 ferritic stainless steel, as shown in Figure 5. Additionally, a constant Poisson ratio of 0.3 was defined.

#### 2.3.4. Process Parameters

As illustrated in Figure 1, the roughing process under study consists of three stages: preheating, descaling, and rolling. The preheating was simulated by defining an initial temperature of the preforms as 35 °C and an ambient temperature of 1163 °C, which are typical for this process. The heating time was set to 200 min. An HTC to air equal to 50 W·m^−2^·K^−1^ was defined.

The descaling phase was simulated with an ambient temperature of 35 °C and a duration of 5 s. An HTC value of 2000 W·m^−2^·K^−1^ was considered appropriate according to the literature [15,16].

The seven passes were simulated using the key parameter values of the rolling schedule provided in Table 1. A friction coefficient of 0.25 was assumed. In terms of thermal conditions, the ambient temperature was set to 35 °C, and the initial temperature of the work rolls was defined as 80 °C. An HTC to air of 20 W·m^−2^·K^−1^ was set [16,17,18]. A roll-gap HTC of 25,000 W·m^−2^·K^−1^ was used during the first three passes [19], where the primary descaling takes place. For the remaining passes, a value of 15,000 W·m^−2^·K^−1^ was defined [18,20].

Regarding emissivity, constant emissivity values in the range of 0.6 to 0.8 [21] have been used in the literature. In the present work, a constant value of 0.8 was used [16], which corresponds with the default value in Simufact Forming and is well within the range mentioned above.

## 3. Results and Discussion

**Figure 6:**
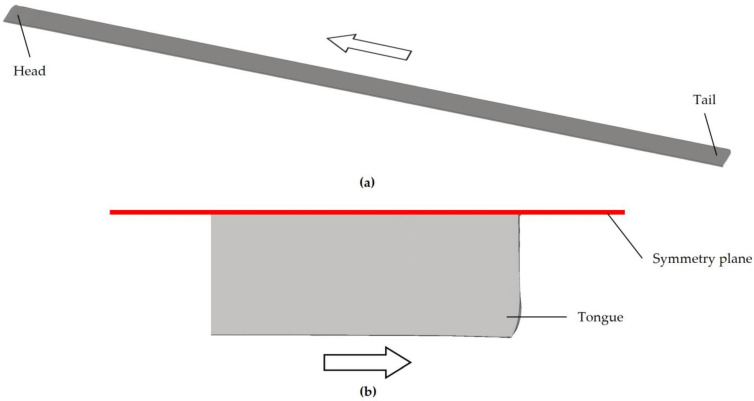
Transfer bar obtained at the end of the seventh pass of the roughing schedule for an AISI 430 ferritic stainless-steel slab. The arrow indicates the rolling direction. (**a**) General view of the simulated transfer bar. (**b**) Top view of the head of the transfer bar, where the rounding of the end, known as a tongue, can be observed.

The model presented in the previous section was employed to simulate the stages of preheating and descaling, and the seven passes of the roughing schedule for an AISI 430 ferritic stainless-steel slab with an initial length of 2000 mm. As a result, a rolled product known as a transfer bar was obtained, as shown in Figure 6.

The obtained transfer bar had approximate dimensions of 16,100 mm in length, 1280 mm in width, and an average thickness of 25.1 mm (Figure 6a). As a result of the rolling process, the length increased by 705%, whereas the thickness of the rolled product was reduced by 87.5%.

Unlike the initial slab, which had a rectangular prismatic shape (Figure 4a), the transfer bar exhibited rounding at the head and tail (Figure 6b). These localized protrusions or extensions that form at the ends of the rolled material are known as tongues. These tongues originate from the non-uniform deformation of the material as it enters and exits the rolls, which may lead to an uneven steel flow. This effect is usually associated with variations in temperature, friction, and material geometry during the rolling process. Tongues may affect the final product quality and, in many cases, require trimming before finishing operations.

**Figure 7:**
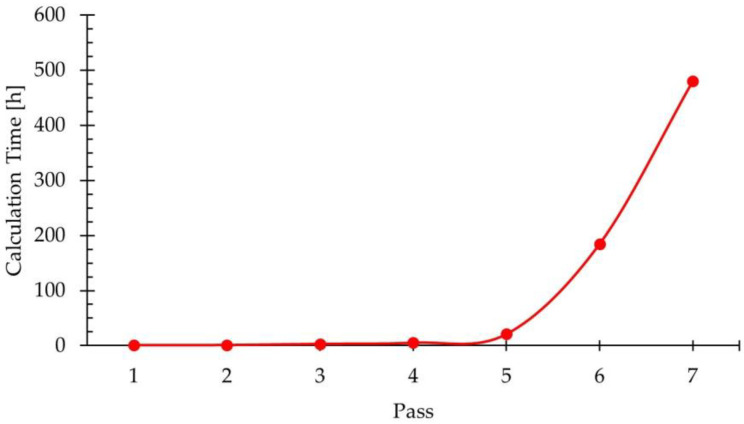
Calculation time used in the simulation of each pass in the rolling schedule of the roughing stage of AISI 430 ferritic stainless steel.

As indicated in Section 2.3.2, the slab was initially meshed using hexahedral elements of 40 mm, such that its thickness was divided into five layers of elements. To maintain the number of elements in the thickness direction, the element size was reduced in each pass. This resulted in a progressive increase in the number of elements in the workpiece for each pass, which significantly affected the calculation times. Figure 7 shows the calculation time required for each of the seven simulated passes in the roughing schedule.

In Figure 7, it can be observed that the evolution of the calculation time shows exponential growth, with a significant increase in the time required to simulate each pass. It is important to highlight that the simulations of the passes discussed in this study were performed using the optimized model in [13], which was simplified in order to minimize the calculation times required to complete the first pass of the roughing rolling schedule. The calculation time largely depends on the number of elements comprising the mesh, which is determined by the element size, and this, in turn, is governed by the thickness of the workpiece. Therefore, the smaller the workpiece thickness, the longer the calculation times. This is particularly relevant in the later stages of the rolling process for flat stainless-steel products, such as hot finishing and cold rolling. In this regard, it should be noted that, despite using the optimized model, the seventh pass of the process required approximately 480 h. This highlights the usefulness of the proposed model, without which performing such simulations would be practically unfeasible. This is evidenced by the absence of studies in the literature that have addressed simulations with characteristics similar to those included in the present work.

### 3.1. Thickness

The employed model enabled the estimation of thickness at various points of the rolled product after each of the seven passes in the studied rolling schedule. Specifically, a grid of equidistantly spaced points in each pass was generated, from which the thickness values were extracted. These values were then used to calculate the average thickness for each pass, along with the standard deviation, as presented in Table 4. Additionally, this table includes the experimentally measured average thickness after each pass.

**Figure 8:**
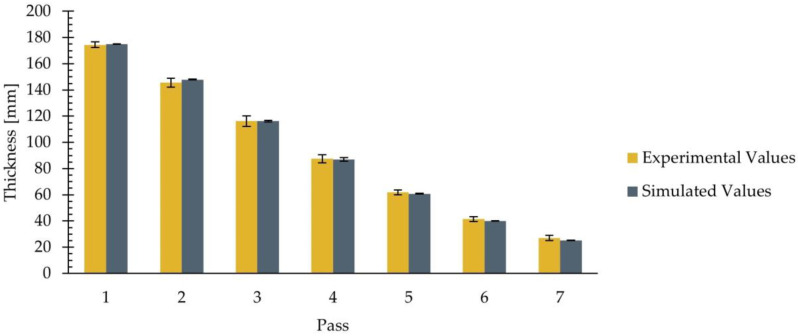
Thickness of the rolled product at the end of each pass. Experimental and simulated average values, with error bars indicating the standard deviations.

Table 4 shows that the simulated average values fall within the interval defined by the experimental standard deviation, indicating a good agreement between the numerical model and the experimental data. To facilitate the interpretation of the results, Figure 8 illustrates the average values of both the experimental and simulated data at the end of each pass, presented in Table 4, with error bars indicating the standard deviation.

Figure 8 clearly illustrates the effect of the rolling process on the thickness of the slab. It can be observed that the thickness of the slab is progressively reduced from the initial 200 mm to 27 mm after the seventh pass. In addition, Figure 8 shows that the average thickness values obtained from the simulation closely match the experimental results. As observed in the figure, the simulated average values fall within the experimental error bars. Furthermore, an overlap between the experimental and simulated error bars is observed, suggesting that there are no significant differences between the simulated results and the experimental data.

**Figure 9:**
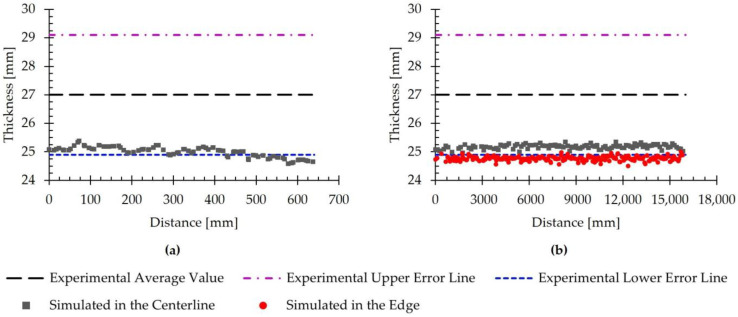
(**a**) Evolution of the simulated thickness at the end of pass seven in the transverse direction, measured at the centerline of the rolled product. (**b**) Evolution of the simulated thickness at the end of pass seven in the rolling direction, measured at the centerline and at the edge of the rolled product. A reference line representing the experimental average value and error lines indicating the standard deviations are included.

Achieving a uniform thickness in the rolled product is one of the primary objectives of the rolling process. For this reason, the uniformity of the thicknesses obtained in the simulated rolled product was analyzed. Figure 9 illustrates the evolution of the simulated thickness of the rolled product in the transverse direction (Figure 9a) and the rolling direction (Figure 9b). In this figure, the experimental average value from Table 4 is also included, along with error lines indicating the standard deviations.

As stated in Section 2.3.1, the simulations were conducted under the assumption that both the geometry and process were symmetric, allowing only half of the slab width to be simulated. Accordingly, Figure 9a depicts the thickness of the rolled product from the center, corresponding to a distance of 0 mm, to the edge, located at approximately 640 mm. In this figure, a slight decrease in the simulated thickness from the center of the rolled material to the edge is observed. In this direction, the predicted values range from 24.6 mm to 25.4 mm, with the highest values observed in the central region of the rolled material and the lowest near the edge. Because the lower experimental error line is positioned at 24.9 mm, certain areas of the rolled transfer bar exhibit thickness values outside the defined interval. These thickness values deviate from the lower limit by only 1.2%, so the error was considered negligible.

Considering the thickness difference observed between the center and the edge of the slab in the transverse direction, Figure 9b represents the thickness variation at these locations along the rolling direction. This figure shows that, both at the center and the edge of the rolled material, the simulated thickness remains approximately constant along the rolling direction, with no significant variations. Additionally, it is clearly observed that, throughout the slab, the thickness at the edge remains below the value measured at the center. Specifically, the simulated values extracted from the center range from 24.9 mm to 25.4 mm, with an average of 25.2 mm. Therefore, the simulated thickness at the center lies within the interval defined by the error bars corresponding to the standard deviation. Regarding the values at the edge, they range from 24.5 mm to 25.0 mm, with an average of 24.8 mm. In this region, most of the simulated thickness values fall slightly outside the interval defined by the standard deviation. Specifically, the average thickness at the edge differs by only 0.4% from the lower limit.

In summary, the obtained results demonstrate the accuracy needed to detect a slight thickness difference between the center and edges in the transfer bar after the seventh pass while maintaining a homogeneous distribution along the rolling direction. Furthermore, there is good agreement between the simulated and experimentally measured values, as the simulated values fell within the limits defined by the experimental standard deviations at almost all points.

No studies were found in the literature comparing the predicted thickness of the rolled material with experimental results. Instead, most studies compared the values of load [22,23], torque [24], or temperature [20,25,26], which were used to validate the simulations. In contrast, this study had access to experimental thickness values for the rolled product after each pass. These values were compared with the simulated results, showing good agreement. Based on these findings, the simulations were demonstrated to be reliable in predicting the thickness evolution throughout the rolling process.

### 3.2. Rolling Load

**Figure 10:**
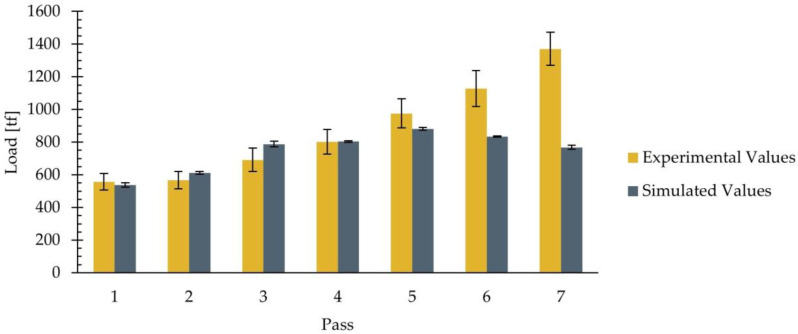
Rolling load in each pass. Experimental and simulated average values, with error bars indicating the standard deviations.

Similar to the thickness data, the proposed model enables the estimation of the rolling load values after each pass. Table 5 presents the average values and standard deviations of the experimental and simulated rolling load, which are shown, along with their corresponding error bars, in Figure 10.

In both Table 5 and Figure 10, it can be observed that the experimental load value progressively increases in each pass. For the simulated values, it can be seen that, in passes one, two, and four, the simulated average values closely match the experimental measurements, with the simulated values remaining within the intervals defined by the experimental standard deviations. In contrast, the simulation slightly overestimated the load in pass three, with the simulated value being 788 tf, whereas the upper limit of the experimental load was 763 tf. This represents only a 3.3% exceedance of the upper limit in terms of relative error. Conversely, in the fifth pass, there was a difference of 0.7% between the simulated value and the lower limit of the experimental load. When comparing the intervals defined by the standard deviations, the lower limit of the experimental interval was 886 tf, whereas the upper limit of the simulated interval was 889 tf, resulting in an overlap between both intervals. Thus, the differences can be considered insignificant. In summary, it can be said that the model used allows for the estimation of load values in the first five passes of the studied rolling schedule with an error percentage lower than 3.5%.

On the other hand, in Figure 10, it can be observed that, in passes six and seven, there was an increase in the difference between the simulated and experimental load values. In these two passes, the simulated average value fell below the lower limit defined by the standard deviation, indicating an underestimation by the model. Specifically, the simulated average value was 834 tf in the sixth pass and 768 tf in the seventh pass. When comparing these values with the lower limits of the experimental value intervals, differences of 18.2% in the sixth pass and 39.5% in the seventh pass were obtained.

As noted in [13], the range of relative error in rolling load predictions varies across different studies in the literature. For example, in [27], Soulami et al. simulated the fifteen passes that constituted the rolling schedule of a flat sample of U-10Mo uranium alloy encapsulated in 1018 steel. When comparing the results of the rolling load with the experimental values, they observed that the predictions started to diverge after the sixth pass, eventually leading to an overprediction of 14% in the final pass. Soulami et al. attributed this divergence to a change in the microstructure of the U-10Mo alloy or the 1018 steel, which resulted in a reduction in flow stress that was not considered in the constitutive relation of the materials.

Wang et al. [28] simulated the hot rolling of AZ31 magnesium alloy sheets. When analyzing the rolling load results, differences of 10–15% between the experimental data and the simulated predictions were observed. According to the authors, this discrepancy was attributed to the omission of the effects of torsion and shear on the dynamic recrystallization of the magnesium alloy.

Phaniraj et al. [16] analyzed the evolution of the rolling load in carbon steel strips through the FEM simulation of six passes in the hot finishing stage. Their results showed that the predicted rolling loads aligned well with the experimental data, with an average relative error of 8% and all cases falling within ±15%. During the simulation, the strip temperature dropped below the equilibrium transformation temperature Ac_3_ before reaching the sixth pass. Furthermore, the predicted rolling loads in the sixth stand matched the measured loads in the mill when the release of retained strain in the workpiece was considered. Additionally, Phaniraj et al. conducted a sensitivity analysis to examine the effect of various parameters on the rolling load prediction. Specifically, changes in the work roll diameter, work roll flatness, work roll temperature, and strip thickness were analyzed. Based on this analysis, the authors determined that these sources of error could influence the rolling load results by up to 10.9% compared to the initial value. These results highlighted the importance of accurately modeling the process under study in FEM simulations of hot rolling, as various factors can significantly affect the precision of the results, particularly the rolling load predictions.

In summary, the results obtained in this study indicate that the employed model allows for estimating the rolling load with an error below 3.5% between passes one and five of the rolling schedule. This error is of the same order of magnitude as that proposed as acceptable in the literature. The estimation error increases to 18.2% and 39.5% in passes six and seven, respectively.

Based on information available in the literature, these discrepancies in the last two passes may be attributed to the model not accounting for potential changes in the microstructure of the material during industrial rolling. As indicated in [13], the constitutive model used allows for the estimation of flow stress by considering the strain, strain rate, and temperature. However, this model does not account for variations in microstructure. According to Phaniraj et al., during the simulation of the process studied by these authors, the material temperature dropped below the equilibrium transformation temperature, Ae3. This factor was not considered during the simulations, which led to a divergence between the experimental and simulated rolling load values. Furthermore, Wang et al. emphasized that, during the hot rolling process, the material is subjected to stresses that may affect dynamic recrystallization. By neglecting these effects, variations in the simulated load results occur, which can lead to discrepancies with experimental data. The conclusions drawn by these authors align with those of Soulami et al., who determined that microstructural changes in the material could lead to modifications in flow stress and that such changes should be considered in simulations to avoid discrepancies in the rolling load results. Consequently, to improve the load estimation, it will be necessary to perform future studies in which a sensitivity analysis of the results is performed to determine the influence of parameters such as the variation in the chemical composition of the AISI 430 stainless steel and their microstructural properties. The analysis should be conducted in the temperature range and plastic deformation conditions characteristic of the process. Based on this analysis, modifications to the constitutive model should be proposed.

### 3.3. Temperature

**Figure 11:**
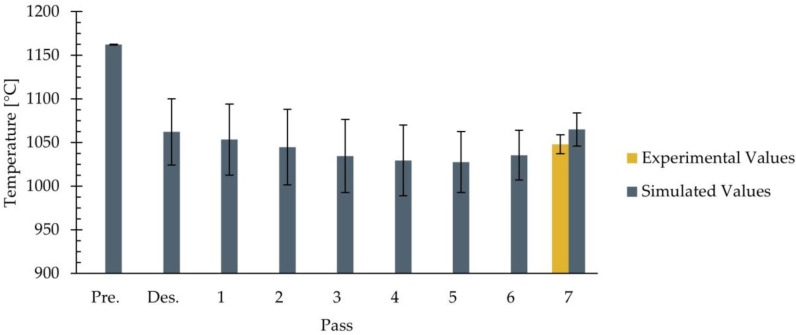
Surface temperature of the rolled product after the preheating and descaling stages and at the end of each pass. Experimental and simulated average values, with error bars indicating the standard deviations.

Another result provided by the simulations is the material temperature. Table 6 presents the average values and standard deviations of the experimental and simulated surface temperatures after the preheating and descaling stages and after each pass of the studied rolling schedule. As shown in this table, only experimental temperature values at the end of the seventh pass of the roughing stage are available. To facilitate the interpretation of the results, Figure 11 illustrates the average values of both experimental and simulated data at the end of each pass, as presented in Table 6, with error bars indicating the standard deviations.

**Figure 12:**
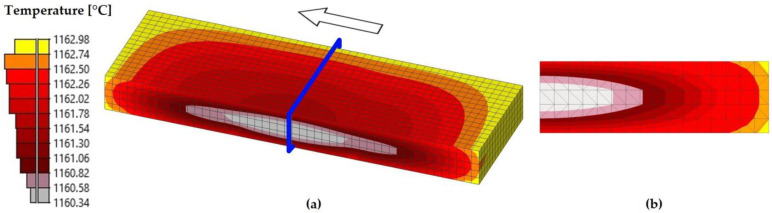
Temperature distribution at the end of the preheating stage simulation. (**a**) Half of the slab. The blue plane indicates the cutting plane used to evaluate the temperature distribution in the transversal direction. (**b**) A cross-section of the slab.

**Figure 13:**
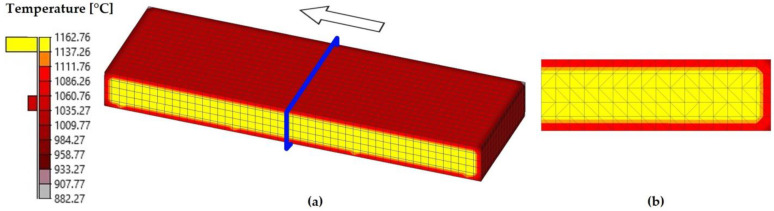
Temperature distribution at the end of the descaling stage simulation. (**a**) Half of the slab. The blue plane indicates the cutting plane used to evaluate the temperature distribution in the transversal direction. (**b**) A cross-section of the slab.

**Figure 14:**
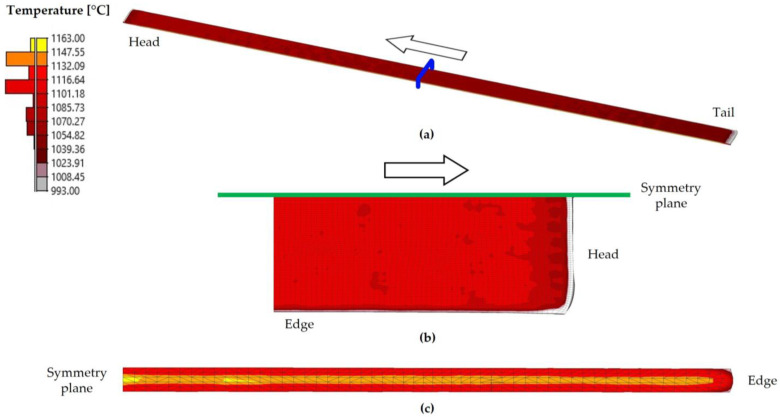
Temperature distribution at the end of the seventh pass of the roughing stage. (**a**) Half of the transfer bar. The blue plane indicates the cutting plane used to evaluate the temperature distribution in the transversal direction. (**b**) Top view of the head of the transfer bar. (**c**) A cross-section of the transfer bar.

As observed in Table 6 and Figure 11, the simulated surface temperature varied throughout the simulated process. During the preheating stage, the slab was heated from ambient temperature to an average surface temperature of 1162 °C, which is close to the furnace setpoint temperature of 1163 °C. Subsequently, in the descaling stage, the average surface temperature decreased to 1062 °C due to the effect of high-pressure water jets. This represents the most significant drop in surface temperature throughout the simulated process. Afterward, during passes one to five, the average surface temperature continued to gradually decrease. Finally, in passes six and seven, an increase in the average surface temperature was observed, which reached values of 1035 °C and 1065 °C, respectively. According to Table 6, the experimental average temperature at the end of the seventh pass was 1048 ± 11 °C. When comparing these values with the simulated average value, a difference of 17 °C was observed with respect to the experimental average value and 6 °C with respect to the upper bound of the error bars, corresponding to differences of 1.6% and 0.6%, respectively. These differences were considered acceptable and consistent with the literature. For instance, in [16], the finishing rolling temperatures were predicted with an accuracy of ±15 °C. Additionally, Figure 12, Figure 13 and Figure 14 show, respectively, the temperature distribution after the preheating and descaling stages and at the end of the seventh pass of the roughing stage.

Figure 12 shows that, at the end of the preheating stage, the temperature was approximately uniform throughout the volume of the slab. Specifically, after the 200 min established for this stage, the surface temperature ranged approximately between 1161 °C and 1163 °C, whereas the internal temperature of the slab was not lower than 1160 °C.

As previously mentioned, at the end of the descaling stage (Figure 13), there was significant variation in the surface temperature of the slab due to the action of high-pressure water jets. Thus, the temperature inside the material reached values of up to 1160 °C, whereas on the surface, it averaged 1062 °C.

The temperature of the transfer bar shown in Figure 14 at the end of the seventh pass exhibited a distribution similar to that observed after descaling (Figure 13). Specifically, the temperature inside the rolled material was close to 1163 °C, whereas the surface temperature decreased to an average value of 1065 °C.

**Figure 15:**
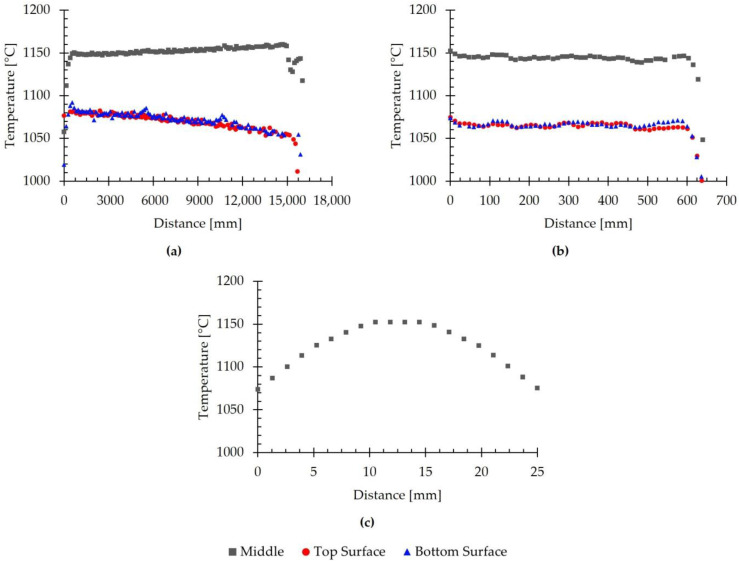
Evolution of the simulated temperature with position, evaluated in different directions, at the end of the seventh pass. (**a**) Evolution in the rolling direction on the top and bottom surfaces and at mid-thickness. (**b**) Evolution in the transverse direction on the top and bottom surfaces and at mid-thickness. (**c**) Evolution in the thickness direction at the center of the transfer bar.

In order to analyze the evolution of temperature in the volume of the rolled material in more detail, Figure 15 shows the evolution of the simulated temperature with position, evaluated in different sections at the end of the seventh pass of the roughing stage. Specifically, Figure 15a shows the evolution in the rolling direction, evaluated at the top and bottom surfaces and at mid-thickness; Figure 15b shows the evolution in the transverse direction, evaluated at the top and bottom surfaces and at mid-thickness; and Figure 15c shows the evolution in the thickness direction at the center of the transfer bar.

Figure 15a shows that the simulated temperature was significantly lower at the center of the transfer bar than on the top and bottom surfaces. This figure also highlights that the temperature at the ends was lower than in the rest of the rolled material for all three evaluated regions. Specifically, the lowest values were primarily located at the head and tail surfaces, reaching as low as 1011 °C at the tail of the transfer bar. For the data collected at mid-thickness, the temperature reached values of up to 1160 °C.

Regarding the evolution of the simulated temperature in the transverse direction, Figure 15b shows that, as in the rolling direction, there was a substantial difference between the temperature at the center and that on the surfaces of the rolled material. In this direction, the simulated temperature remained roughly constant in the central region of the transfer bar, with an average value of 1065 °C on the top surface, 1067 °C on the bottom surface, and 1144 °C in the interior. Near the edges, the temperature decreased, reaching minimum values of 1001 °C, 1006 °C, and 1048 °C, respectively.

On the other hand, Figure 15c shows that the simulated temperature in the thickness direction was the highest at the center of the transfer bar, reaching 1153 °C. Moving toward either the top or bottom surfaces, the simulated temperature progressively decreased until reaching the surface, where a temperature of 1075 °C was reached. This is consistent with what was observed previously.

The simulated temperature results at the end of the seventh pass are similar to those presented in [13] for the first pass, showing similar behavior in all three directions for the different evaluated regions. Thus, the highest temperatures were recorded at the center of the rolled product, whereas the lowest temperatures were concentrated at the surface. Consequently, a thermal gradient was observed near the surface of the rolled material.

Upon reviewing the literature, similar cases were found. For instance, in [16], Phaniraj et al. obtained a linear gradient of 40 °C between the surface and the center of the rolled material. Meanwhile, [29] observed a difference of 40–50 °C between the surface and the center when simulating the hot rolling of steel slabs.

Based on the obtained results, it is concluded that the numerical model used could predict the temperature evolution of the material in the simulation of the initial stages of the industrial hot rolling of large-scale AISI 430 ferritic stainless-steel slabs. This model enabled the prediction of the temperature distribution at different stages and the analysis of temperature variation between the surface and the interior of the simulated material. The predicted temperature results at the end of the last pass were close to the experimental values, with a deviation of 0.6%. The obtained results indicate that there are significant temperature differences between the surface and edges of the transfer bar compared to its center. This could lead to microstructural variations that may affect the performance of these regions. Future studies will involve experimental investigations aimed at detecting potential performance differences that could be explained by the temperature variations identified in the simulations.

### 3.4. Effective Plastic Strain

**Figure 16:**
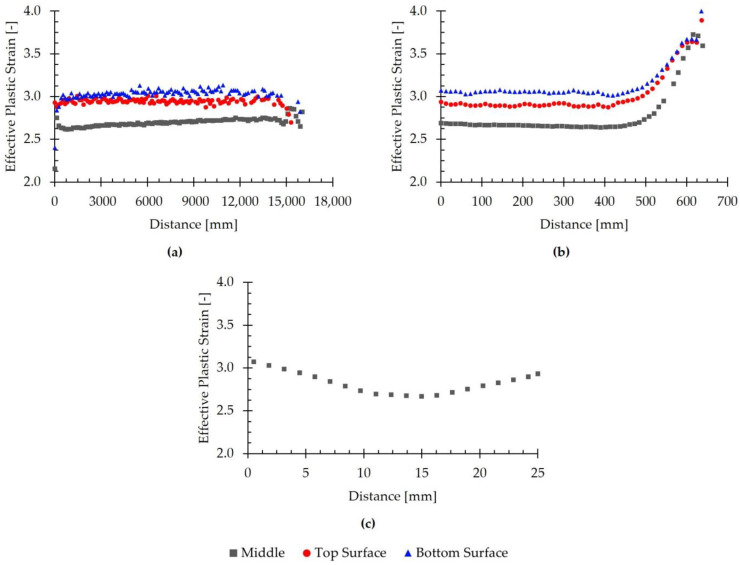
Evolution of the simulated effective plastic strain with position, evaluated in different directions, at the end of the seventh pass. (**a**) Evolution in the rolling direction on the top and bottom surfaces and at mid-thickness. (**b**) Evolution in the transverse direction on the top and bottom surfaces and at mid-thickness. (**c**) Evolution in the thickness direction at the center of the transfer bar.

The strain of the transfer bar at the end of the seventh pass was analyzed, as shown in Figure 16. This figure presents the evolution of the simulated effective plastic strain as a function of position, evaluated in the rolling direction on the top and bottom surfaces and at mid-thickness (Figure 16a), in the transverse direction on the top and bottom surfaces and at mid-thickness (Figure 16b), and in the thickness direction at the center of the rolled material (Figure 16c).

Figure 16a shows that the effective plastic strain was higher on the top and bottom surfaces than in the core of the transfer bar. Specifically, the average values were 2.94 on the top surface, 3.03 on the bottom surface, and 2.69 in the interior of the rolled material. Additionally, this figure shows that, in all the evaluated regions, the equivalent plastic strain remained approximately constant along the rolling direction, with no significant differences observed between the head, center, and tail of the transfer bar.

The analysis of the effective plastic strain distribution in the transverse direction, as shown in Figure 16b, revealed that the simulated values remained approximately constant in the central region of the transfer bar. From the center of the transfer bar, corresponding to 0 mm in the figure, up to approximately 400 mm on either side, the average values were 2.90 for the top surface, 3.06 for the bottom surface, and 2.66 for the interior of the rolled material. Beyond this point, the effective plastic strain increased towards the edges, where the maximum values were reached in all three regions. Specifically, the strain values at the edges were 3.89 for the top surface, 4.00 for the bottom surface, and 3.59 for the interior.

Figure 16c shows that the evolution of the effective plastic strain in the thickness direction is consistent with the trends observed in the other two directions. The highest values were found on the top and bottom surfaces, measuring 2.93 and 3.07, respectively, whereas the lowest value, 2.67, was recorded at the center of the transfer bar.

The effective plastic strain results obtained in this study at the end of the seventh pass of the roughing stage are similar to those reported in [13] for the first pass. As stated in Part 1 of this study, the results obtained are consistent with those found in the literature. McLaren and Sellars [30] investigated the hot rolling of 316L stainless-steel slabs and found that the simulated strain exhibited an increasing trend with distance from the slab center, which was attributed to the effect of redundant shear deformation. Zhang et al. reported in [31] the presence of a shear deformation process that tends to localize near the strip surface during the hot rolling of an unlubricated 304 stainless-steel strip. In [32], Xiangyu et al. analyzed the deformation behavior and bonding characteristics of a Cu/Al laminated composite plate. Their findings indicated that the strain on the free surfaces of the Cu/Al plate was greater than that in the core region, where the two materials were bonded. This phenomenon was also reported by Jiang et al. in [33] during the simulation of the hot rolling of a clad plate composed of Q235 carbon steel and 1Cr13 stainless steel. Their study revealed that the effective strain increased from the core, where the two materials were bonded, toward the free surface. The authors noted that, as the rolling process advances, the near-surface region undergoes greater deformation compared to the core, resulting in a more pronounced deformation of the surface metal. Sun et al. [34] simulated a multi-pass hot rolling process for flat 7A04 aluminum alloy products. Their results showed a non-uniform effective strain distribution, with higher values at the surface than in the interior of the material. According to the authors, central region movement is restricted, limiting material flow and resulting in lower strain values inside. In contrast, surface metal flows more easily due to friction with the work roll, which enhances strain and increases temperature, further promoting deformation. Thus, surface metal exhibits greater flowability during hot rolling.

The simulation results are consistent with those found in the literature. Our study demonstrates a non-uniform effective plastic strain distribution, with higher values at the free surfaces and lower values in the core. This gradient arises from restricted material flow in the central region and enhanced deformation at the surface due to friction-induced heat. Such behavior, observed across various materials and rolling conditions, confirms the validity of the simulation approach.

### 3.5. Equivalent Stress

**Figure 17:**
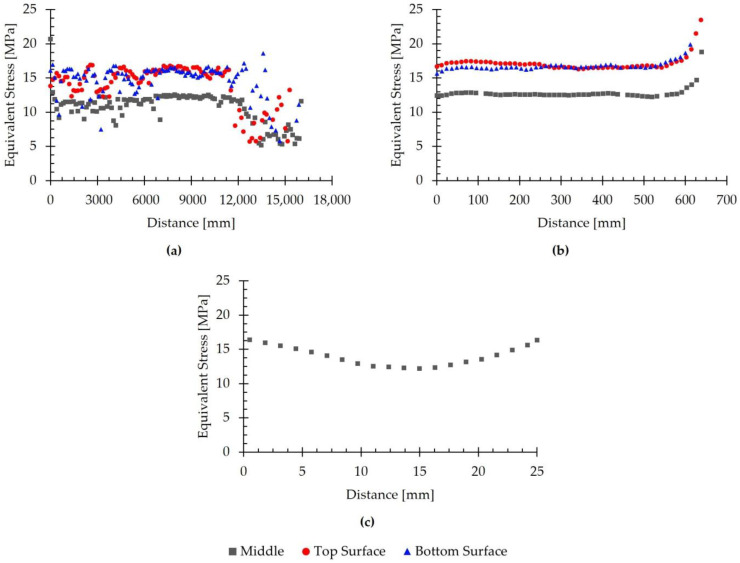
Evolution of the simulated equivalent stress with position, evaluated in different directions, at the end of the seventh pass. (**a**) Evolution in the rolling direction on the top and bottom surfaces and at mid-thickness. (**b**) Evolution in the transverse direction on the top and bottom surfaces and at mid-thickness. (**c**) Evolution in the thickness direction at the center of the transfer bar.

The simulations performed also made it possible to estimate the values of the equivalent stress. Figure 17 shows the evolution of the equivalent stress as a function of position, evaluated at the end of the seventh pass for different directions. Specifically, the analysis was conducted in the rolling direction on the top and bottom surfaces, and at mid-thickness (Figure 17a); in the transverse direction on the top and bottom surfaces, and at mid-thickness (Figure 17b); and in the thickness direction at the center of the transfer bar (Figure 17c).

When analyzing Figure 17a, no clear trend in the simulated values can be determined. At the head and tail of the transfer bar, the values appeared to fluctuate arbitrarily. In contrast, in the central region, the equivalent stress remained approximately constant across the three evaluated zones. This figure also shows that, for most of the length of the bar, the equivalent stress was higher at the surface than in the interior. Specifically, the average simulated values on the top and bottom surfaces were 14.1 MPa and 14.7 MPa, respectively, whereas the average value in the interior of the rolled material was 10.7 MPa.

By contrast, in Figure 17b, a clear trend in the equivalent stress can be observed in the transverse direction. Similar to in the previous case, the equivalent stress was comparable on the surfaces of the rolled material and was higher than in the interior. For the three evaluated regions, the stress remained approximately constant in the central region, between 0 mm and 500 mm, with an average value of 16.9 MPa on the top surface, 16.6 MPa on the bottom surface, and 12.6 MPa in the interior of the material. Near the edge, the stress increased noticeably, reaching a maximum value of 23.5 MPa on the top surface.

Similarly, the results presented in Figure 17c show that the lowest equivalent stress value was obtained in the interior of the rolled material, reaching 12.2 MPa. As the distance to the top or bottom surfaces decreased, the simulated stress value increased, reaching 16.4 MPa. These results are consistent with the observations in Figure 17a,b.

The simulated equivalent stress distribution exhibits a behavior similar to that previously observed for the effective plastic strain distribution. As mentioned in Section 2.3.3, the constitutive equation used to model the material in the simulations was a temperature-dependent viscoplastic analytical model that incorporates strain hardening and thermal softening effects. Thus, because the strain on the free surfaces of the rolled material is higher than that observed in its interior, the equivalent stress follows the same trend. However, as mentioned in Section 3.2, the rolling load results for the final passes of the roughing stage suggest that the model does not faithfully reproduce the behavior of AISI 430 steel studied experimentally. This could influence the simulated equivalent stress values in passes six and seven. Therefore, although these results are consistent with findings reported in the literature, they should be interpreted with caution. Future studies should analyze the influence of microstructural and compositional changes in AISI 430 stainless steel under process-specific temperature and deformation conditions to refine the constitutive model.

## 4. Outlook

In summary, the proposed model enables the complete simulation of the roughing stage in the hot rolling process of large-format AISI 430 ferritic stainless-steel slabs. This model allows for the simulation of an industrial rolling schedule without relying on laboratory-scale simplifications, two-dimensional models, or small-scale samples. Through the implementation of remeshing strategies and adaptive meshing, the model successfully captures the large-scale processes implemented in industrial multi-pass rolling. Furthermore, the optimization strategies applied in [13] contribute to maintaining relatively low computational times. The model effectively replicates industrially observed phenomena, such as the formation of tongues at the head and tail of the rolled material. Moreover, it enables the accurate prediction of slab thickness for a given reduction profile, as well as the temperature distribution throughout the volume of the rolled product. Additionally, it provides reliable estimations of the rolling load and offers insights into the distribution of strain and stress, yielding results consistent with those reported in the literature.

Despite the optimizations and simplifications implemented in [13], computational times remain high in the final passes due to the increased number of elements required to preserve the resolution in the thickness direction. This issue is particularly relevant for simulating subsequent stages of the rolling process while maintaining the full length of the workpiece. If simulating the finishing stage of the studied process, the model would need to accommodate workpieces with thicknesses as low as 3.5 mm and greater lengths than those considered in the present study. Further research is required to evaluate this scenario and, if necessary, develop strategies for efficiently simulating such products. Another limitation is that while the model accurately predicted the rolling load up to the fifth pass, it failed to provide satisfactory predictions for the sixth and seventh passes, showing significant deviations from the average experimental values. As discussed previously, this discrepancy could be attributed to limitations in the constitutive material model, which may not adequately describe the behavior of the material at the high strain levels observed in the final passes.

Future research will focus on refining the constitutive model or exploring alternative models to improve the prediction accuracy in the final passes of the roughing stage. Additionally, further efforts will be directed toward reducing computational times for the final passes. Another aspect to be investigated is the sensitivity of the simulation results to variations in key rolling schedule parameters. The input parameters used in the simulations correspond to average values employed at the Acerinox Europa S.A.U. facilities for a specific grade of AISI 430 ferritic stainless steel. However, in industrial practice, these parameters may fluctuate around their nominal values, potentially affecting the final product. Consequently, future studies will analyze the influence of variations in the reduction ratio, rolling speed, edge roll gap, and material temperature at furnace exit on the simulation results. Once these aspects have been addressed, the industrial-scale finishing stage can be numerically simulated.

## 5. Summary and Conclusions

In this study, a previously validated and optimized numerical model from [13] was used to simulate the initial stages of the hot rolling of large-scale AISI 430 ferritic stainless-steel slabs. Using this model, preheating and descaling, and seven passes of the roughing stage of an industrial rolling schedule were simulated. The commercial finite element software Simufact Forming was employed. The simulations provided results for the thickness of the rolled material; rolling loads; and distributions of temperature, effective plastic strain, and equivalent stress. The results can be summarized as follows:

- The finite element numerical model used enabled the simulation of the preheating and descaling, and the seven passes of the roughing stage in the industrial rolling schedule of large-scale AISI 430 ferritic stainless-steel slabs. Through the simulation, a transfer bar with an approximate length of 16,100 mm, a width of 1280 mm, and an average thickness of 25.1 mm was obtained.
- The transfer bar obtained from the simulation exhibited rounding at both the head and tail. This phenomenon, known as tongues, is common in the hot rolling of flat products as a result of non-uniform deformation in these regions.
- It was observed that the calculation time required to complete the simulation of each pass increased exponentially. This was due to the reduction in the thickness of the rolled product, which led to a decrease in element size in order to maintain five layers of elements in the thickness direction. Consequently, the number of elements comprising the mesh of the workpiece increased, directly impacting the calculation time.
- The average values of the simulated workpiece thickness were in agreement with the experimental data in each of the studied passes. When analyzing the thickness distribution in the final pass, the simulation allowed detecting slight variations in thickness between the centerline and edges of the transfer bar.
- Regarding the rolling load, the results indicate that the employed model allowed estimating the load with an error below 3.5% in the first five passes. In contrast, the relative error in passes six and seven was 18.2% and 39.5%, respectively. After reviewing the available literature, the authors determined that the observed differences could be attributed to the existence of slight differences between the stainless steel used experimentally and that used as a model. Therefore, it will be necessary to further study these differences with the final objective of refining the constitutive equation used in the simulations.
- The surface temperature results at the end of the seventh pass resemble the experimental data, with a difference of 0.6%. When analyzing the temperature distribution, gradients were observed near the surface of the workpiece. The highest temperatures were found in the interior of the material, whereas the lowest temperatures were located on the surfaces.
- A non-uniform effective plastic strain distribution was obtained, with higher values at the free surfaces and lower values in the core. According to the literature, this gradient results from restricted material flow in the central region and increased surface deformation due to friction-induced heating.
- The simulated equivalent stress exhibited a distribution similar to that of the equivalent plastic strain, with higher values on the free surfaces of the material and lower values in the interior.
- Temperature, plastic deformation, and equivalent stress differences were observed at the edges of the rolled product. These differences could lead to anomalous behaviors in these regions of the material, and their existence will be confirmed in future work through experimental studies.

## Figures and Tables

**Table 1:** Average values of the key parameters used in the roughing stage of the hot rolling of AISI 430 ferritic stainless-steel slabs. Data provided by Acerinox Europa S.A.U.

Pass	Initial Thickness [mm]	Reduction Ratio [%]	Work-Roll Gap [mm]	Edge-Roll Gap [mm]	Linear Velocity [m·min^−1^]
1	200.0	12.8	175.2	1269.0	156
2	174.5	16.6	147.9	N/A	180
3	145.6	20.2	116.2	1253.0	196
4	116.2	24.8	87.0	N/A	214
5	87.4	29.3	60.7	1253.0	270
6	61.8	33.0	39.9	N/A	279
7	41.4	34.8	24.9	1268.0	245

N/A: not applicable.

**Table 2:** Values of the main mechanical and thermal properties of AISI 430 at various temperatures close to the working conditions. Data provided by Acerinox Europa S.A.U.

Temperature [°C]	Thermal Expansion Coefficient [°C^−1^]	Thermal Conductivity [W·m^−1^·K^−1^]	Specific Heat Capacity [J·g^−1^·K^−1^]	Density [kg·m^−3^]
800	13.05·10^−6^	24.83	0.73	7704
900	13.69·10^−6^	26.62	0.70	7480
1000	14.24·10^−6^	27.84	0.69	7440
1100	14.55·10^−6^	28.74	0.69	7400
1200	13.97·10^−6^	28.91	0.69	7360
1250	N/D	30.88	0.70	7310

N/D: not determined.

**Table 3:** Main parameters of the initial mesh for the slab, the rolls, and the pusher.

Part	Diameter [mm]	Length [mm]	Width [mm]	Thickness [mm]	Element Type	Element Size [mm]
Slab	N/A	2000	640	200	Hexahedral	40
Work roll	940	800	N/A	N/A	Tetrahedral	50
Edge roll	910	500	N/A	N/A	Tetrahedral	50
Roll of the roller conveyor	400	800	N/A	N/A	Tetrahedral	50
Pusher	N/A	800	400	100	Tetrahedral	50

N/A: not applicable.

**Table 4:** Average values and standard deviations of the experimental and simulated thickness after each pass of the studied rolling schedule. Experimental data provided by Acerinox Europa S.A.U.

Pass Number	Experimental [mm]	Simulated [mm]
1	174.5 ± 2.1	175.1 ± 0.2
2	145.6 ± 3.4	147.9 ± 0.3
3	116.2 ± 4.1	116.2 ± 0.6
4	87.4 ± 3.2	86.9 ± 1.3
5	61.8 ± 1.8	60.8 ± 0.3
6	41.4 ± 1.9	39.9 ± 0.1
7	27.0 ± 2.1	25.1 ± 0.2

**Table 5:** Average values and standard deviations of the experimental and simulated rolling load after each pass of the studied rolling schedule. Experimental data provided by Acerinox Europa S.A.U.

Pass Number	Experimental [tf]	Simulated [tf]
1	557 ± 51	538 ± 14
2	567 ± 52	611 ± 9
3	691 ± 72	788 ± 18
4	802 ± 75	804 ± 5
5	975 ± 89	880 ± 9
6	1128 ± 109	834 ± 4
7	1371 ± 101	768 ± 12

**Table 6:** Average values and standard deviations of the experimental and simulated surface temperatures after the preheating and descaling stages and after each pass of the studied rolling schedule. Experimental data provided by Acerinox Europa S.A.U.

Stage/Pass Number	Experimental [°C]	Simulated [°C]
Preheating	N/A	1162 ± 1
Descaling	N/A	1062 ± 38
Pass 1	N/A	1053 ± 41
Pass 2	N/A	1045 ± 43
Pass 3	N/A	1034 ± 42
Pass 4	N/A	1029 ± 40
Pass 5	N/A	1028 ± 35
Pass 6	N/A	1035 ± 29
Pass 7	1048 ± 11	1065 ± 19

N/A: not available.

## Data Availability

The original contributions presented in this study are included in the article. Further inquiries can be directed to the corresponding author.
